# Unconscious Motives and Actions – Agency, Freedom and Responsibility

**DOI:** 10.3389/fpsyg.2018.02777

**Published:** 2019-02-21

**Authors:** Christoph Lumer

**Affiliations:** Dipartimento di Scienze Sociali, Politiche e Cognitive, Università degli Studi di Siena, Siena, Italy

**Keywords:** unconscious motive, unconscious action, responsibility, freedom, agency, intentional causalism, new unconscious, priming

## Abstract

According to many criteria, agency, intentionality, responsibility and freedom of decision, require conscious decisions. Freud already assumed that many of our decisions are influenced by dynamically unconscious motives or that we even perform unconscious actions based on completely unconscious considerations. Such actions might not be intentional, and perhaps not even actions in the narrow sense, we would not be responsible for them and freedom of decision would be missing. Recent psychological and neurophysiological research has added to this a number of phenomena (the “new unconscious”) in which behavior is completely unconscious or in which the decision or its execution is influenced by unconscious factors: priming, automatic behavior, habitualized behavior, actions based on plain unconscious deliberations, intrusion of information from the dorsal pathway, etc. However, since this makes up the largest part of the behavior which is generally regarded as action, intentionality, yet agency, responsibility and even compatibilist freedom of decision for the largest part of our behavior may be threatened. Such considerations have led to a lively debate, which, however, suffers from generalizations that lump all these unconscious phenomena together. In contrast, the aim of this article is to discuss individual unconscious influences on our behavior separately with respect to what extent they require changes in traditional conceptualizations. The first part (sections 2–4) of the article outlines the “traditions” and their elaborations: the intentional causalist concept of action, an associated empirical theory of action and standard concepts of responsibility and compatibilist freedom of decision, as well as the challenges for them. In the second part (sections 5–9), the aforementioned unconscious influences on our actions (except for automated and habitualized actions, which I discuss elsewhere) are examined: 1. unconscious priming, 2. dynamically unconscious motives, 3. dorsal pathway information influencing conscious decisions, 4. unconsciously altered execution of conscious intentions, and 5. unconscious deliberations and decisions. To what extent do these phenomena C1. require a change in the concept of action, C2. curtail intentionality or agency, C3. responsibility, and C4. freedom? The result is: The curtailments prove to be far less dramatic than they initially appear; they require more watchfulness but no conceptual change.

## 1. Topic and Structure of the Article

This article deals with a challenge to the traditional conceptions of action and intentionality as well as of responsibility and freedom represented by findings in psychology about the pervasive influence of unconscious factors on our actions. Already Freud (1901–1924/1941, 1923/1940) assumed that a large part of our decisions is influenced by unconscious motives or that we perform unconscious actions based on completely unconscious thoughts which are hidden from our consciousness and conscious control because of their rejected content. Such actions are possibly not intentional, perhaps not even actions in the strict sense, and we might not be responsible for them. The Freudian unconscious is the dynamic, i.e., motivated unconscious. Recent psychological research, however, has revealed a number of further and pervasive phenomena, the new unconscious (Hassin et al., 2005), where behavior is executed completely unconsciously for mere “technical” reasons of effectiveness and efficiency or where the decision is influenced by unconscious factors as a by-product; hence it is a non-dynamic unconscious without any critical motives. Phenomena of this sort include: priming, actions based on unconscious deliberations, implicit motives, etc. Some examples are: after having been unconsciously primed on stereotypes of elderly people, subjects walk slower; subjects unconsciously primed for achievement make more efforts to perform a task well; subjects throwing darts at pictures of persons they like have worse scores as subjects aiming at pictures of neutral faces; unconsciously seizing the opportunity and opening the window; or after being unconsciously primed on ‘impression formation’ subjects, instead of simply reading a list of words referring to personal features, make up their mind about what a person with these qualities would be like. Automatic actions – such as changing gears when driving a car, unconsciously imitating the conversation partner, unconsciously executing a (simple) intention when the right moment has come – are another part of the new unconscious.

The existence and knowledge of these phenomena could imply several theoretical and practical problems or challenges. *C1. Decision-theoretical model of action:* Actions could function completely differently than usually assumed. In particular, there may be no central controlling instance that weighs up the reasons for action, takes a decision according to the strongest reasons, which in turn causes the action. *C2. Agency:* Possibly, the traditional intentional-causalist concept of action – according to which actions are caused by intentions – is no longer applicable, since there are no (or hardly any) actions at all (in this sense). *C3. Responsibility:* The domain of what we, as conscious subjects, really control may shrink to a tiny size, and thereby the sphere for which we are responsible may diminish accordingly. *C4. Freedom:* If actions are not consciously chosen on the basis of our reasons for action, or if their selection no longer depends on what we consider important, then it could be that we do not even have a compatibilist freedom of decision (in the sense of a rational, authentic or autonomous decision), and perhaps not even freedom of action in the conditional sense.^1^ So the mentioned discoveries are not only challenges for the traditional conception of action but also for our legal and everyday handling of responsibility and freedom.

The aim of this article is to discuss these challenges posed by the psychological findings about unconscious motives and actions. The mentioned phenomena are not only very different, they also have different implications regarding agency, responsibility and freedom; therefore, they must be examined individually. More specifically, 1. the interference of various unconscious mechanisms (e.g., priming and dynamically unconscious motives) in conscious decisions, 2. alterations in the execution of actions by unconscious mechanisms, and 3. several types of unconscious deliberations and decisions are examined, if and to what extent they influence or should influence C1: the decision-theoretical model of action, C2: agency and intentionality of the behavior, C3: responsibility for it and C4: freedom of decision. Automatic and habitualized behaviors, i.e., learned, schematic and automatically triggered and executed behaviors, are also unconscious but make up a big separate and particular group. I will not deal with them here; I have discussed them in a parallel article again with regard to the challenges C1 to C3: Lumer (2017). Implicit bias (see e.g., Brownstein and Saul, 2016) is also not discussed here because of its peculiar problems. – Executionary details of our actions are usually not intended, but are controlled mostly automatically (to a large extent even by different brain areas than our decisional structures), and therefore, also unconsciously. These phenomena are not discussed here either, because they are not problematic for and do not conflict with the traditional conceptions of action and responsibility inasmuch as they do not threaten action-control by our ego.

In the next three sections (sections 2–4) I will explain the challenged philosophical background, i.e., a decision theoretical model of action, the intentional-causalist model of action and intentionality, traditional criteria of retrospective responsibility and criteria of freedom of decision, in somewhat more detail and how it may be challenged by the psychological findings. Then, in the second part of the article (sections 5–9), I will give an overview of the three mentioned groups of unconscious phenomena in action (1. unconscious influences on conscious decisions, 2. unconscious alterations in the action execution, and 3. unconscious decisions), systemize and explain them, and assess whether and how they contribute to the four challenges. Because the phenomena themselves are sometimes disputed, and above all since only few and not always convincing explanations for them are available, I have made some effort to provide my own explanations of the examined phenomena.

## 2. The Challenge of Unconscious Motives and Actions for the Traditional Conceptions of Actions and for Freedom

The most widely accepted, classical conception of action is *intentional causalism*: An *action* is a behavior caused [in a non-deviant way (Lumer, 2008)] by a respective intention, which represents this behavior beforehand. In order to be an action also in a narrow, *emphatic sense*, the intention, additionally, has to be “really ours,” namely volitive, i.e., a (in principle) rationalizable integration of our conscious attitudes towards the action (Lumer, 2013). The philosophical reason for intentional causalism, apart from its (arguable) empirical reality, is that it explains the value of actions, namely that actions conceived in this way give practical power to our conscious ego:^2^ make it control our behavior and thereby change pieces of the world for realizing our desires and implementing the decisions of our reason (ibid.). The intentional-causalist conception has not only been dominant in the history of philosophy – from Aristotle via Hume, Kant to, e.g., Davidson – it is also prevalent in everyday thinking of actions, and it is the basis of criminal, civil as well as moral responsibility and hence also of the criminal justice system. Intentional causalism is not the only theory of action proposed in philosophy. But here I can neither discuss the competing conceptions nor justify intentional causalism (but see: Lumer, 2005, 235–250; Lumer, 2010, 967; 969–970; Lumer, 2013, 511–517). However, for the main task of this article it is sufficient that intentional causalism is the most important conception of action in philosophy as well as in everyday thinking and it is the basis for common Western conceptions of moral and legal responsibility. If all this were called into question by the above-mentioned empirical findings, then this would already generate sufficiently important problems to justify investigating whether this questioning really exists. The same applies to the following sketches of the concepts of freedom and responsibility.

In order to be able to respond to the mentioned psychological challenges, beyond the bare minimum of intentional causalism some general features of a fitting model of decision making are needed. Here I will make recourse to my *optimality belief theory of intention and decision making* (Lumer, 2005), which says that intentions consist in optimality beliefs that a certain option is the best among the considered alternatives; furthermore, it assumes a process of decision making, leading to such optimality beliefs, with some affinities to the processes presupposed in rational decision theory but allowing much more flexibility: for example, the optimality theory assumes that there is a vast spectrum of possibilities of how extended the underlying deliberation is, from immediately believing that a certain option is optimum, over considering only one option other than doing nothing with only one relevant advantage – this is similar to Aristotle’s practical syllogisms –, to, at the other extrem, constructing complex options, compiling relevant consequences, assessing their probability and desirability, etc. Optimality judgments can also remain implicit: the subject searches for information that enables him to make an optimality judgment about one of the alternatives and keeps track of which important information is still missing. When he has collected all the necessary information, then he believes in the judgment of optimality without having to consciously represent it. – With the help of this theory one can also explain more precisely what *intentionality* is according to the “simple view” (intentionality presupposes a corresponding intention) (Adams, 1986): An *action* is *intentional* under a certain description, iff this description was used in the intention and the action was not caused in a deviant way (on deviance: Lumer, 2008). An action *consequence* is *intentional* under a certain description iff (I1) the agent has considered it under this description in his compilation of consequences, (I2) this consequence had the prominent role of an aim or means in his planning, and (I3) the action produced this sequence in a non-deviant way. An action *consequence* is brought about only *knowingly* iff conditions I1 and I3 are fulfilled, but not I2. – There is no need to take exactly this optimality belief theory to be able to reply to the psychological challenges. However, in philosophy of action there is hardly any further elaborated empirical deliberation model, and the sketched model will be sufficient.

The unconscious influences on our decisions and actions mentioned above may also impair or even nullify our *freedom of decision*. For one thing, freedom of decision can be conceived *compatibilistically*, so that the determinacy of our decisions does not exclude their freedom. The three most important conceptions of compatibilistic freedom of decision are: 1. According to *(subjectivist) rationalism*, the aim of a free decision is to find the best option and initiate its implementation. The best option is the one that fulfills the maximum of our desires. To this end, good options for action must be constructed, the relevant consequences of these options must be found and evaluated according to our desires (e.g., Dennett, 1984; Smith, 1997; Lumer, 2002). Philosophical (in contrast to economic) theories of subjective rationality also require that desires be critically filtered in one way or another (e.g., Brandt, 1979, 10–16; 70–89; 110–129; Lumer, 2000/2009, 241–427; 521–548; Lumer, 1998). 2. According to *theories of autonomy*, decisions are free if they choose actions according to whether they arise from the essence or core of the person (e.g., Frankfurt, 1971, 1999; Ekstrom, 2005). This presupposes that in deliberation relationships are established between the subject’s firmly anchored desires and values and the action. 3. *Objectivist theories of good reason* require that a free decision be sensitive to objective reasons, i.e., recognizes reasons for and against the various options and chooses the action according to these reasons (e.g., Nagel, 1986; Wolf, 1990). The nature of such objective reasons, our subjective access to them and their existence are controversial. However, if there are such reasons, their consideration in the decision definitely requires some cognitive complexity – as Kant’s illustrations of free decisions according to the Categorical Imperative show (Kant, 1785/1903, BA 18–19; 53–57; 66–69 = A.A. 403; 421–424; 429–430) – which probably is only achieved by slow, conscious cognitive processes. – For another thing, freedom of decision can be conceived *incompatibilistically*. Incompatibilists generally see indeterminacy as a necessary but not sufficient condition for freedom of decision. Even if most incompatibilists do not specify sufficient conditions, the most plausible proposals for this, again, go in the direction of the just-outlined compatibilist theories. So we can limit the further discussion to these three theories.

Before presenting and discussing the conditions for retrospective responsibility, it is convenient to first explain the challenges for the three theories and their applications by the unconscious influences outlined above in more detail. We must distinguish between two fundamentally different challenges: (i) theoretical challenges, i.e., possible refutations of the empirical components of the theories presented, and (ii) practical, normative challenges, i.e., more or less profound and widespread limitations of the action character of our behavior, its intentionality, our freedom of decision and (to be discussed below) responsibility.

(i) *C1: Optimality belief theory and C2: intentional causalism:* The discovered unconscious influences on our actions and decisions could be evidences that the whole idea of intentional causalism and the empirical theory elaborating and extending it, i.e., the optimality belief theory, are completely false (or at least empirically nearly empty). Actions could function completely differently in that the “decision” is taken on an unconscious level, and our conscious deliberations and intentions, e.g., only inform us about what has been decided “there”; this might be called the “*display thesis*” about our consciousness. [Libet (1985; more references and critique: Lumer, 2014a) and Wegner (2002; critique: Lumer, 2014b) suggest this, Koch and Crick (2001) defend it, and philosophers like (Andy) Clark et al. (2013, 1–30) radicalize it.] Apart from the empirical confutation, in such a case the traditional conception of action would be empirically void; there would not be any control of our behavior by our conscious ego and our conscious desires; neither would there be freedom of decision, freedom of action or any responsibility via our conscious assessment and respective control of possible actions. Here I will not discuss the display thesis in general (but see: Lumer, 2014c, 7; 10–12) since it is much too far-fetched. What should interest us here instead is whether the empirical phenomena discussed below perhaps confirm the display theory or falsify the optimality judgment theory. – *C1: Optimality belief theory:* The optimality belief theory holds, among others, that at least in complex deliberation the final optimality judgment is reached by integrating the various aspects of the options. Precisely because of this complexity, the integration must be conscious – the unconscious cannot take so many aspects into account correctly –; and in order to be taken into account at all in the conscious integration, the aspects must be conscious. That unconscious factors influence a conscious deliberation, as it was observed with various of the mentioned phenomena, should then actually not occur at all; it would be prima facie a refutation of the optimality belief theory or at least require considerable explanations. Moreover, this should lead to incorrect integration of the various aspects of the decision – although the decision and action usually look quite rational. – *C2: Intentional causalism:* Some of the phenomena in question regard unconscious influences on our execution of actions. These unconscious influences intervene between the intention formation and the execution of the action, thereby leading to a distortion of the action and possibly to a misalignment of intention and action. Pervasive and strong unconscious influences of this type could question intentional causalism as an empirical theory since one cornerstone of this theory, i.e., the regular correspondence of intention and behavior, would no longer hold. – *C3: Freedom:* Since the theories of freedom outlined above are not empirical but normative, they cannot be confuted by the empirical findings to be discussed here. However, the confutation of the sketched empirical theory would imply normative challenges (to be discussed below).

(ii) *C2: Intentional causalism and agency, C3: freedom, C4: responsibility:* Some of the empirical findings suggest that the intention itself – or a quasi-intention, if we do not want to call it “intention” – can be unconscious along with the process leading to it (i.e., perhaps an unconscious deliberation) as well as the action itself. We might even be aware of the resulting behavior but would not recognize it as our *action* – as for example in the case of what psychoanalysis conceives as a Freudian slip. In such a case all the conditions of an action, as they are established by intentional causalism, might even be fulfilled, the mental conditions, however, only by unconscious mental states. Whether the resulting behavior would still be an action is debatable; in any case it would not be an action in the emphatic sense since it would not be controlled by our conscious ego. And without this control the decision and action would not be free and we could not be responsible for the action. Similar considerations hold if single motives or whole parts of the deliberation remain unconscious: As far as these parts influence the action and thereby the action’s consequences, these actions and action consequences are not intentional in the narrow sense. They are alienated from us because we do not recognize them as originating from our person; therefore, our decisions in these respects would not be free either. – *C2: Agency, intentionality, C3: freedom, C4: responsibility:* If unconscious factors intervene between our intention and its execution and thus lead to a misfit of the action to the intention the executed behavior would not be under our control; hence we would not be responsible for it – at least not directly –; still less would we have acted freely.

## 3. The Necessity of Consciousness for Emphatic Agency, Intentionality and Freedom

Why and to what extent exactly do the emphatic character of action, intentionality in the narrower sense, freedom and responsibility require consciousness? Are the presented (and still to be presented) normative conceptions not too narrow? What are the relative advantages of consciousness? The value of good decisions is that they identify and implement the really best action. As they are theorized in rational decision theory, the steps to achieve this are:

*D1: Compile options:* The relevant, i.e., possibly optimal, options must be identified or, in the case of complex and in any respect novel situations or goals, must first be newly constructed. In order to be possibly optimum, the options must fit to the action situation.
*D2: Identify consequences:* All possible relevant consequences of these options must be identified.
*D3: Determine probabilities:* The probability of these possible consequences must be determined.
*D4: Evaluate consequences:* The desirability of these consequences must be determined ultimately on the basis of the fundamental evaluation criteria of the agent and taking into account critical filters.
*D5: Integrate information:* The information acquired in the previous steps must be integrated into an optimality judgment based on criteria such as expected desirability or prospect desirability.

These steps do not have to take place in exactly this order. And in fact, for mere economic reasons, they are only carried out in such detail and completely in exceptional cases; practical rationality requires very specific shortcuts (Lumer, 1990, 390–400; Lumer, 2005, 241–247; 248–250). However, to prevent these shortcuts from possibly leading to dramatically wrong decisions, certain safeguarding conditions must be fulfilled. Above all, there are two characteristics of unconscious information processing, because of which the unconscious mind can perform each of these steps correctly in simple cases only: (i) a high degree of modularity, i.e., the restriction to specific tasks on the basis of specific information without including any other possibly relevant information, and (ii) associativity, i.e., information processing according to semantic links without taking syntax or formal criteria into account (Levy, 2014a, 40–47). Conversely, conscious information processing does not have these limitations: (i) According to the global workspace theory (Baars, 1988, 1997; Dehaene and Naccache, 2001; Dehaene et al., 2011), consciousness of information serves precisely to make this information universally available to large parts of the brain, so that these parts, when the information is relevant to them, can react to it in a specific way. By making a thought conscious, in particular thoughts on a decision situation, a certain option or a possible consequence, the thought is so to speak globally published in the brain [Levy calls this “broadcasting” (Levy, 2014b, 62–69)] and thereby exposed to all sorts of comments and criticisms from all relevant parts of the brain (Lumer, 2014a, 86–87; 96–100). (ii) By means of conscious information processing, we are able to process non-automated algorithms neatly, in particular to check compliance with complex criteria or to proceed constructively step by step, for example in arithmetical operations.

(i) Of the above-mentioned deliberation steps, compiling options (D1) and identifying consequences (D2) now contain very strong generalizations: all relevant options and all relevant consequences must be determined; or more precisely, rationally considering our epistemic restrictions, all relevant options and consequences that are somehow accessible to us with the current knowledge must be determined. The options can in turn be composed from smaller steps, and consequences can also be determined by chaining consequences of consequences. All this requires the use of very diverse information scattered over many modules, which can only be retrieved from the global workspace. This is the role of consciousness as a global workspace in the active search for options and consequences. – Unconscious routines usually stop and reawaken attention when unusual situations occur. Only the conscious search for and construction of flexible alternatives with the help of the global workspace can then initiate the adequate coping with the situation. The same applies to the discovery and exploitation of special opportunities for action. – Let us now return to the shortcuts. With the very largest shortcuts, we believe or presume to have immediately found the plausibly best option or at least a good approximation of it; further considerations would probably only make this option more expensive. Even in this case of the simplest decision, consciousness still has the function of broadcasting the (imminent or just made) decision to all relevant parts of the brain, thus exposing it to comprehensive criticism and thereby possibly receiving feedback that there are relevant and perhaps significantly better options or, that there are further possibly relevant consequences, in particular negative side effects, which make it advisable to suspend the decision for the time being.^3^

(ii) However, if they are to be correct, the other deliberation steps – determining probabilities (D3), evaluating consequences (D4), integrating information (D5), but in part also the first two (e.g., when the feasibility of an option has to be checked or conditional or remote consequences have to be determined inferentially) – require the processing of algorithms or the checking of compliance with criteria. Only by conscious processing can this be achieved with sufficient accuracy^4^.

What does all this mean on the normative level? *Emphatic agency* and *freedom in the sense of autonomy* are lacking with unconsciousness, above all because the decision cannot take all of the subject’s essential concerns into account (D2) and also the filter function is switched off in the evaluation (D4); hence the decision cannot represent the subject as a whole.^5^ Unconsciously aspired goals are not pursued *intentionally in the narrow sense*, because while unconsciously believing to bring about the respective action consequence, the subject does not know in a somewhat more comprehensive sense what to bring about this consequence means, i.e., what it implies, which total desirability it has, etc. *Freedom of decision in the sense of rationality* is lacking in unconscious decisions and decisions with unconscious components, because the criteria for rational decisions are only met by coincidence; most detailed deliberations with all steps D1 to D5 cannot be made unconsciously anyway. And even for fulfilling the standards of the simplest but sometimes still rational decision, the unconscious decisions lack the precaution that the option in question is exposed to spontaneous criticism in the global workspace regarding better options and relevant side effects.

## 4. The Challenge of Unconscious Motives and Actions for Responsibility

The following list of conditions for retrospective moral and (in Western societies) also approximately criminal responsibility – which aim at efficient social control of human actions via, among others, punishments and rewards (Lumer, 2012) – is an attempt at an ethical systematization of all conditions widely accepted though not brought together in the literature and which are roughly present also in most everyday thinking on retrospective responsibility. Hence, the list does not take up the particular conditions of unorthodox or minority positions like versions of incompatibilism about responsibility, the doctrine of double effect, guidance control, ascriptivism or attributionism.

A subject *s* is (retrospectively) *responsible* for his action *a* (that *s* does *A* at time *t*) <for the event *e* produced by *a*> iff:

*R1: Objective deed component:* That *s* does *A* at *t* is an action. <And this action has produced *e*.>.
*R2: Attenuated principle of alternate possibilities: s* could have acted otherwise (namely, if *a* is immoral or forbidden *s* could have performed a morally better/permitted action *b*), if *s* had not been prevented from doing so by over-determination. In the context of possible unconscious influences, “*s* can do *B*” means: If *s* intends to do *B*, and (in case of risk of unconscious mental distortions) uses standard procedures known in his society to exclude psychic distortions, then *s* does *B*.
*R3: Subjective deed component/mens rea: s* does *A*<has produced the consequence *e* by his doing *A*> 1. premeditated, 2. goal-intentionally, 3. means-intentionally, 4. knowingly, or 5. culpably unknowingly (cf. Kenny, 1978, 1–2; 5–7). These subconditions become weaker and weaker from R3.1 to R3.5. Which of these five subconditions to apply depends on the type of action or consequence: The more serious the consequence, the weaker the subjective part of the act can be in order to imply responsibility. Hence, in the case of socially very harmful actions, culpable ignorance alone is sufficient for responsibility.
*R4: Sanity, soundness of mind:* 1. The intention to *a* must originate from considerations of *s*, e.g., the intention may not be caused by hypnosis; 2. at the decision time the ability of *s* to deliberate must not be below a level critical for the inclusion of morally and legally relevant information; specifically *s* recognizes alternatives to *a*, and recognizes the possible consequences of *a* which are also immediately recognizable for others and are highly relevant for the decision, *s* evaluates them according to his evaluation criteria and integrates these evaluations into an overall assessment, which in some way takes into account all consequences of importance – possibly only by deliberately ignoring them.
*R5: No shielding of responsibility by other persons’ responsibility: s*’ decision for *a* was not so pre-structured by others – by a structuring of 1. the decision situation or 2. the internal conditions of the decision: evaluations, factual assumptions, assumptions about alternatives – that it was subjectively imperative for *s*. 1. So there was no real coercion, compelling incitement, action ex ufficio, no order or the like (Duff, 1990, 83) and 2. neither was there any brainwashing, targeted misinformation, nor (futuristic) neurological change of the foundations of the valuation or similar factors.
*R6. Reasonableness:* Not to do *a* (or a substantially similar act *a*^∗^) would have been reasonable for subject *s*.

The conditions for the responsibility for consequences of action should actually also take into account the possibility of producing consequences jointly. The conditions for responsibility for omissions and for events which could have been prevented by omitted actions are also missing here. With regard to the problem of consciousness, however, these cases do not differ from what is presented here. Since conditions R1, R3.1, and R6 are irrelevant for the discussion of the significance of unconscious influences on our actions for responsibility they will not be discussed any further.

*On R2, alternate possibilities:* If someone has the intention to carry out a certain action but unconscious factors intervene on the way from this intention to the desired goal – during the formation of the implementation intention or during the execution itself – so that the actually desired action is not executed, does this mean that the subject could not carry out the actually intended action? The term ‘*s* can do *B*’ used in the condition of alternate possibilities in a first attempt may be interpreted conditionally, following G.E. Moore (1912, ch. 6): ‘If *s* intends/decides to do *B*, then *s* does *B*.’ According to this simple interpretation, however, the fact that the unconscious distortions prevented the execution of the originally intended action would already be the proof that the subject could not perform this action – so that the subject might no longer be responsible for his action and its consequences. But this simple interpretation relieves the subject of responsibility too quickly. If the agent foresees or suspects the influence of unconscious distortions, then he could use self-control mechanisms to achieve the desired result nevertheless. One such strategy to eliminate unwanted unconscious influences is to proceed strictly analytically in the decision, in case of major and important decisions also by writing, i.e., to note down the individual steps (alternatives, consequences, impact assessment) and in particular to calculate the overall values. Condition R2 then provides that, in order to correctly say that the subject could not perform this action, he would also have failed during execution if he had used such control mechanisms [analogous considerations for akrasia: Kennett (2001), 155].

*On R3, mens rea:* The above formulations of *mens rea* still leave open whether the knowledge or the intention can also be unconscious. Is unconscious belief or intention sufficient for knowingness or intentionality? The sense of *mens rea* in the overall concept of responsibility and punishment is: Only if the agent believes that he brings about the (incriminated) consequence *e* can he be socially discouraged from bringing it about, (i) namely by his recognizing the moral or social significance, i.e., the moral value, of *e* and, because of its negativity, abandoning the action or (ii) by at least recognizing that this action or the bringing about of this consequence is punishable, so that bringing it about will probably lead to his punishment (Lumer, 2012, 708–711; 714–718). The threat of punishment is of no use with people who do not even believe that they will bring about the incriminated consequence, and their subsequent punishment cannot have a deterrent effect, because the threat of punishment can only lead to the prevention of action if the subject can associate the action – by believing in this consequence – with the threat of punishment. However, both ways of preventing subject *s* from committing the act imply that he draws further inferences beyond the mere knowledge of the consequences, that in his deliberation he also spontaneously recognizes certain consequences of the consequence and takes them into account in his decision. However, this spontaneous recognition of certain consequences of the consequence is a typical task that can only be solved in the global workspace, i.e., by consciousness.^6^

*On R4: Sanity, soundness of mind:* The conditions for sanity and insanity are generally formulated only very vaguely in the criminal law literature^7^ or very concretely via the existence of certain conditions such as strong affect, severe alcohol intoxication, delusions, mental illness. Hence, they do not specify the general nature of the limitation of the ability to deliberate. The above conditions, instead, are intended to be general, but nevertheless reasonably precise and address the functions of deliberation. What is particularly interesting in the context of the discussion of the significance of unconsciousness for responsibility, – after excluding responsibility for merely unconsciously aspired or believed consequences (R3) – is mainly: If unconscious assumptions of consequences, which *per se* are not morally or legally relevant, massively influence the agent in his conscious decision and mislead him, so that the conscious integration of decision-relevant information is wrong, does this reduce his soundness of mind? An agent, e.g., unconsciously believes that if he does the (forbidden) action *A*, namely stealing and drinking an extremely expensive wine, he will be cured of a bad disease; then he decides to do *A* for conscious but insignificant, rather weak reasons, say, to want to try how such a wine tastes, although he knows that doing *A* is forbidden. In such examples, the agent is not obviously pathological. But he does not include in his conscious deliberation reasons for action which are actually decisive and arrives at, if one takes into account the unconscious reasons, a comprehensible but, if one only considers the conscious reasons, completely wrong integration of these reasons, an irrational decision. In the case of wine theft, these distortions are so great that not even awareness of the forbiddance of his actions and the known threat of punishments prevent the perpetrator from his action. Soundness of mind is intended to guarantee that the agent has at least a minimum of practical rationality in order to be able to react adequately to the moral significance of the situation and the options. In the example, this level is undercut. Serious unconscious distortions often lead the agent to look at the decision again, consciously and critically, and then revise it. The lack of such an “awakening” is an indication of the subject’s currently insufficient rational sovereignty.

*R5: No shielding:* If condition R5 is not fulfilled, i.e., if someone else has structured the decision situation in such a way that the decision is pre-programmed, then the other person is responsible and no longer the subject. Such shielding may exist especially in the case of targeted priming.

## 5. Conscious Decisions Influenced by Unconscious Priming

Now I will analyze various modes of unconscious influences on our decisions and actions and discuss their critical relevance with respect to the four challenges. I distinguish three main groups of such influences according to the stage when this influence applies. Then several types of influences have to be distinguished within these groups. The groups and mechanisms are:

1. A conscious decision is influenced by an unconscious mechanism:
     1.1. unconscious priming (this section);
     1.2. dynamically unconscious motives (section 6);
     1.3. dorsal pathway information (section 7);
2. the execution of a conscious decision is altered by an unconscious mechanism (section 8):
     2.1. unconscious priming;
     2.2. resistances;
3. the decision itself is unconscious (section 9):
     3.1. plain unconsciously deliberated action;
     3.2. unconscious decision with dynamically unconscious motives;
     3.3. sleepwalking.

So, let’s start with the first group: A conscious decision is influenced by unconscious priming. Here “unconscious priming” means that the *influence* of the prime is not noticed by the agent; the prime itself can be unconscious, e.g., subliminal, i.e., presented so briefly (and often later covered) or parafoveal (immediately outside of the central area of our vision) that it cannot be consciously detected; but it can also be conscious. A standard example is priming for high achievement by achievement-relevant words. The subjects in the first part of the experiment have to resolve a word-search puzzle, viz. to find in a 10 × 10 matrix of letters 13 words, which were listed under the matrix. In the achievement priming, the list of the 13 words to be searched for included: “win,” “compete,” “succeed,” “strive,” “attain,” “achieve,” “master.” This is the priming part. In the second part of the experiment the subjects again had to resolve a word-search puzzle, namely they had 10 min each time to find as many as possible of ten hidden words, from an indicated semantic field – foods, bugs and colors – in three 10 × 10 letter matrices. None of the subjects suspected any influence of the first part of the experiment on the second. Nonetheless on the average, subjects primed with the achievement words found 26 words, the control group only 21.5 words [Bargh et al., 2001, experiment 1 (1016–1017)].

At least these were Bargh’s results. Since the 1980s Bargh is the most prominent researcher on unconscious priming effects on conscious decisions and on the execution of actions. More recently, however, his studies have been in the center of the replication crisis in social psychology: Other researchers have tried to replicate many of Bargh’s experiments and very often did not succeed; among the experiments whose replication failed were also some of the most spectacular like “achievement priming” (Bargh et al., 2001, experiments 1 and 3; failed replication, e.g., Harris et al., 2013) and the “Florida effect” (Bargh et al., 1996, experiment 2; failed replication: Doyen et al., 2012; general overview of replication problems of Bargh’s experiments: Bartlett, 2013). Other researchers suspect that the causes of the failed replications are statistical problems – small samples, weak effects, low significance threshold – and confirmation bias – the experimenters were able to manipulate the results in the direction of the hypothesis. – Within the discussion of the present article, a first possible strategy for dealing with this unclear empirical situation would be to say: The whole phenomenon of priming is not proven at all; presumably it is an artifact; then it cannot be a challenge for the conceptions of action, freedom and responsibility defended here. So, one would not need to discuss the phenomenon further here. If, however, later it were to become clear that several of the effects are real, i.e., that unconscious priming of decisions and action executions exists, then the theories discussed here would have an open flank. Therefore, I will pursue a different, more cautious strategy in the following: A great many priming effects have been found; it cannot be ruled out that they are *all* artifacts, but this is unlikely. *Which* of them are artifacts cannot be established at this point. But I expect (or at least do not exclude) that there are some priming effects that work qualitatively like the ones reported, even though some or many examples from the literature may be artifacts. So, according to this strategy, the *nature* of the priming effects is taken seriously – but not necessarily the individual example –; and the mechanisms behind them are accepted as challenges requiring discussion and comment from an action-theoretical and ethical point of view. This discussion follows immediately. The topic of this discussion is then, for example, Bargh’s *theory* on the *mechanisms* behind the priming effects, not the individual example. And the aim is to show that these mechanisms are much more conscious than Bargh assumes and thus mostly leave room for agency, responsibility and freedom. If, contrary to expectation, it should turn out one day that all *types* of priming effects are artifacts, this would not be detrimental to the theories defended here, because then the empirical basis of the challenges for these theories would not exist in the first place.

Let us apply the second strategy to the experiment on achievement priming! I do not find the authors’ explanation of this effect very plausible;^8^ therefore, I present you my own attempts at an explanation: *i.* The achievement priming induces an *atmosphere of achievement*, which includes feeling powerful and a desire to act correspondingly with much energy and high performance. This achievement atmosphere brings about that only very effective search strategies pop up in the subject’s conscious mind when he thinks about how to approach the search task; and he may simply choose the only or first of these as suitable for finding many words. This explanation does not presuppose that the subject strives for achievement in a narrow sense, i.e., wants to be the best and to follow the most effective strategy; but the achievement atmosphere lets effective search strategies come to mind first, as if the subject had the achievement goal and had sought for effective search strategies. *ii.* In a variant of this explanation the priming induces only the *achievement desire*, which is sufficient to reduce the range of options that spring to mind to highly effective ones. *iii.* Finally, in a situation where some effort is required, the still existent activation of the achievement words may directly induce the occurrence of a desire for high performance and the respective *conscious goal* setting, which in turn leads to a search for very effective strategies.

This was only one example of conscious decisions influenced by unconscious priming with conscious primes; there are many others (e.g., Fitzsimons and Bargh, 2003, studies 1 and 4a; Bargh et al., 2001, experiments 3, 4, and 5; Bargh et al., 1996; Dijksterhuis and van Knippenberg, 1998, 868–873). Similar effects have also been obtained by subliminal priming (e.g., Custers and Aarts, 2007; Pessiglione et al., 2007; more examples: Custers and Aarts, 2010, 49–50). Apart from the already mentioned ways in which priming works (i.e., inducing a certain emotional and evaluative atmosphere, inducing the activation of a general desire, inducing the formation of conscious goal), I also propose the following pathways: *iv. Priming of an option salience:* Priming can directly induce that certain options of a later opened option set are neuronally activated or become salient for the subject. This implies that the subject can choose this option for different reasons, though for the observer, at least at the statistical level, it seems to be a decision for the very specific contentual reason connected to the priming content. Hence, there may be an *extensional* decision for high achievement, but no intensional decision with this content. *v. Priming of a framing:* Priming may induce the subject to feel situated in a certain environment, or to feel a certain kind of reality, e.g., a hostile or cooperative environment, as normal; and this leads to some shifting of the subject’s behavioral or evaluative standards. *vi. Priming of a role:* Similarly, the subject may be primed to feel to be in a certain role. *vii. Priming of a specific aspect of a choice:* Priming may also induce that certain aspects of the options are intrusive and hence more often and more strongly considered in the decision. For many examples of the literature several of these explanations are possible.

More generally unconscious priming of conscious decisions seems to function as follows. During deliberation we have to search for possible options and their possible consequences. This is an association or inspiration task. For such tasks it is not surprising and quite normal that present neuronal activations, including subliminally caused activations, induce semantically connected ideas to pop up and, through their intrusiveness, to have more weight in the decision. The latter possibility is opened primarily by the fact that a comparative desirability quantification of the options’ relevant aspects often is not possible or is relatively difficult and costly and, therefore, not undertaken so that the pondering of these desirabilities is done in an estimating holistic value judgment, where present activations may have their corresponding influences. The priming effects will work unnoticed by the subject especially if the relevant options are roughly or even exactly of equal value for him so that a shift of desirability and a distortion of the decision are not critical; most experiments in the priming literature work with such choices (where the alternatives for the subjects are rationally of roughly equal value) (Di Nucci, 2014, 41–42); hence it is not surprising that priming can easily influence a decision. Primed irrational desirability shifts in favor of clearly worse options probably would be noticed and then consciously corrected accordingly.

What does this analysis of the functioning of unconsciously primed conscious decisions imply for the four challenges? *C1. Optimality judgment theory of deliberation and intention:* All described mechanisms lie within the scope of the optimality belief theory. The optimality judgment making up the decision rests on the consideration of advantages and disadvantages of the option; that this judgment is not really made inferentially but only in an estimating holistic way is not excluded by that theory. *C2. Agency and intentionality:* The resulting actions are also actions in an emphatic sense. In contrast to what many researchers on priming at least insinuate, the decision in these cases is taken consciously and the intention is executed normally. Furthermore, the decision reflects the subject’s conscious desires – though their weights sometimes may be somewhat distorted, however, in the usual range of desirability judgments without precise criteria. *C3. Responsibility:* With respect to responsibility it is important that the decision process be conscious and hence under critical supervision. Strong distortions with respect to the subject’s values and standards of rationality would probably be noticed and corrected. And if instead of fostering options within the subject’s usual range, the priming brings strange or disliked options – e.g., criminal acts – to mind, these options are still consciously evaluated. If the holistically made optimality judgment is distorted by priming, these distortions in the examples, however, are comparatively small, within the normal range and far from violating the quite broad condition of soundness of mind (R4). Therefore, in these cases there is no infringement of the subjects’ responsibility. *C4. Freedom:* Priming does not impute extraneous desires to the subject, censored desires are not helped to break through, existing own desires are not hindered, and options are taken from the normal repertoire (though with a bias for certain options) and then subjected to conscious valuation, etc. Therefore, autonomy is not restricted. And as long as the distortions of the holistic judgment of optimality remain within the scope of the usual, the rationality of the decision is not called into question by the unconscious influences.

## 6. Dynamically Unconscious Motives Influencing Conscious Decisions

The second group of unconscious influences on conscious decisions consists of dynamically unconscious motives as studied by Freud: The respective motives play a role in the decision, but because of resistances against their delicate contents they are suppressed from access to consciousness and are accessible to the agent’s consciousness only after a psychoanalytic revelation.

An example in kind described by Freud is: When restructuring his consulting room, a neurologist finds a simple old wooden stethoscope, which he does not need any longer. After thinking for a while where to place it he puts it laterally on his desk so that it sits exactly between his own and the patient’s chair. At the beginning of the psychoanalysis of this event the neurologist says he could have put the stethoscope in other places. A long analysis of the neurologist’s career aspirations, however, reveals that on the one hand the stethoscope represents the successful satisfaction of his sexual desires by the vicinity to his objects of love during auscultation and on the other the separation from his patients: The stethoscope stands between him and his patients like Sigurd’s sword between Sigurd and Brunhilde (Freud, 1901–1924/1941, 216–219). The later revelation of the unconscious motives’ influence on the decision and the description of their contents completely relies on the subject’s ideas. The decision itself is conscious. And the preceding deliberation will have included many other conscious considerations (not reported by Freud) like the stethoscope’s decorative and symbolic value. The characteristic feature of this case (apart from the fact that the effective intention is conscious), however, is the mixture of conscious and unconscious motives; the unconscious motives are present only in the form of intuitive inclinations, without conscious justification and without revealing their real content. From Freud’s description it is not clear how much the unconscious desires influenced the decision at all. The resulting action looks rational even without these unconscious motives, but, as the neurologist himself says, it lies in a spectrum of (almost) equivalent alternatives, so that the unconscious motives may well have been the deciding factor. My proposal about how the integration of conscious and unconscious motives can take place in the conscious decision – after all the neurologist consciously integrates only his conscious considerations into the conscious decision – is similar to that given for the functioning of unconscious priming: The agent does not execute a precise inference from the various (vague) desirability considerations to the optimality judgment, i.e., a kind of arithmetical calculation; instead he estimates the total desirabilities of the options, with an eye on all the aspects taken into consideration, in holistic value judgments, whereby the decisive steps are made unconsciously. And this gives room for the unconscious motives and for their influence going unnoticed by the agent. The difference with respect to what happens in priming is that dynamic unconscious motives are really *desires*, which might change the option’s total desirability. As pertinent desires, during the deliberation they should have been consciously considered and exposed to critical scrutiny and rational assessment on a par with the desires for the other consequences of the object: are they legitimate or perhaps irrational (e.g., because they are unfulfillable), what is the total desirability of their fulfillment? The results of this scrutiny and assessment should then be integrated into the total evaluation of the options. Instead, because of their delicate content, these desires are suppressed in the consciousness and are integrated unfiltered into the overall evaluation.

A fictitious example [based on (Levy, 2014b, v)] may illustrate the more extreme and critical possibilities of this mechanism. The president of a search committee rejects a candidate for a job, because he unconsciously reminds her of her ex-husband; the only other candidate is clearly less qualified; and having much authority, her vote is decisive. If one disregards possible irrational desires (such as punishing the ex), the unconscious desire could be not to want to have people with these traits around or in important positions. The conscious deliberation and public justification is done in such a way that the president exaggerates the rejected candidate’s minor defects, belittles his achievements, whitewashes defects of the worse candidate and overstates his achievements. But this is only a retrospective rationalization, while the main mechanism is again that already before, in a holistic judgment, the representative of the ex-husband is devalued as unacceptable. The subsequent vote is intentional and probably also rational: the president has voted in favor of what she considers to be the better, but is, in fact, clearly the worse candidate. This is ignorant wrongdoing. The critical point, however, is the preparatory action for forming judgments about the candidates. The judgments’ aim was to determine the objectively best candidate. This goal was missed because of the unconscious influences. So could the president not carry out the action of finding the best candidate? She could have done the evaluation analytically, and then the distortions would probably have been noticed; hence, she could make a correct evaluation, the alternate possibility condition (R2) was fulfilled. With such important decisions as in the example, it is also a duty of care to take measures to exclude distortion, even if there is only a suspicion that one’s own assessment could be distorted. Because the president has not complied with this duty of care before, her later voting action is a culpably ignorant wrongdoing (R3.5).

How is the influence of dynamic unconscious motives to be assessed from the practical philosophical viewpoints? *C1. Optimality judgment theory of deliberation and intention:* What goes on in these cases is covered by the optimality belief theory of intentions in a similar way as unconscious priming of conscious deliberations. However, there is a characteristic mixture of conscious and unconscious aspects and valuations which are integrated in the conscious optimality judgment. *C2. Agency and intentionality:* The resulting behavior is an action in the broad sense caused by a respective intention. However, the emphatic agential character is more or less reduced: The decision is inter alia also determined by desires that are not part of the conscious ego. The unconscious desires (in Freud’s example: to be close to one’s patients but at the same time to be kept away from real intimacy by a symbolic boundary) also only result in unconscious intentionality. This prevents the action from being an action in the emphatic sense. The president, instead, achieves her unconscious goal of not wanting her ex-husband’s representative to be around her, etc., but misses her conscious goal of identifying and hiring the best candidate. Therefore, which aspects of the action are intentional must be determined in each individual case. *C3. Responsibility:* Responsibility must also be judged on a case-by-case basis when conscious decisions are influenced by dynamically unconscious motives. Responsibility also depends on moral or legal duties of care and on the possibility of acting otherwise. So Freud’s neurologist is responsible for his conscious action and the achievement of his conscious goal, but he is not responsible for the realization of the unconscious intention, because it was not conscious and no corresponding duty of care existed. The president, instead, is also responsible for the realization of her unconscious intention, because she has violated a compliable duty of care. With strong neuroses it may be impossible to act differently even with the help of self-control strategies (R2); the conscious consideration may also be so distorted that the neurotic is insane (R4). *C4. Freedom:* In any case, the influence of dynamic unconscious motives on the decision curtails freedom of decision, because these motives evade the potential censorship by the conscious ego, so that the agent no longer controls which of her desires enter into the decision. Also, the integration of the information into the optimality judgment (D5) is always distorted, thus impairing rationality. But these curtailments vary in degree according to the case; with the president, they are so severe that they even impede reaching the conscious goal.

## 7. Conscious Decisions Influenced by Dorsal Pathway Information

Information from the actually automatic processing of perceptions via the dorsal pathway can also unconsciously influence conscious decisions. One example is: The experimenter pours sugar into two bottles on which the subjects had to affix, according to their own choice, the labels 1 “not sodium cyanide, not poison” with a red skull and crossbones preceded by the word “not,” and 2 “sucrose, table sugar.” The experimenter then placed one cup of beverage in front of each of the two bottles and added one spoonful of sugar from the corresponding bottles to the cups. The subjects then had to choose from which cup they wanted to drink. They were relatively reluctant to drink from the cup marked “not sodium cyanide, not poison” (Rozin et al., 1990). Substantially, the two alternatives are completely equivalent. Rozin explains the preference with an interference of dorsal processing of perception information, which actually serves the automatic action control, in particular fine-tuning, into the conscious decision. Dorsal, unconscious processing is encapsulated and merely semantic, thus ignoring the syntax; hence “not sodium cyanide,” etc., is perceived as “cyanide,” etc. This false interpretation then influences the conscious decision. – According to Rozin’s explanation, the unconscious information would intervene directly in the formation of the optimality judgment, thus distorting it. This would correspond to how the influence of unconscious priming on a conscious optimality judgment was explained above. But the subjects may also have a conscious, irrational residual doubt, which they even recognize as false, but from which they can hardly escape. Then the unconscious influence is to be located in the formation of the belief about the possible toxicity; the subsequent decision itself would even correspond to the criteria of a rational decision: the integration of desirabilities and probabilities into the optimality judgment would be completely correct. In any case, the influence of unconscious false information is relatively small; it only comes into play relatively strongly because the two alternatives are actually completely equivalent.

*C1–C3:* Not only is the last piece of the pathway along which unconscious dorsal information influences conscious decisions just the same as in unconscious priming (section 5), our normative evaluation is also almost the same. *C4:* However, there is one difference: the *rationality* of the subjects who preferred not to drink from the cup labeled “no cyanide” is curtailed, insofar as they can certainly recognize that their reservations are completely unfounded but apparently cannot detach themselves from them. However, because of the actual equivalence of the options, this is no real loss; and we may again surmise that significant distortions would have been noticed by the subjects and consciously corrected by them.

## 8. Unconsciously Altered Execution of Conscious Intentions

After a conscious (proximal) intention has been formed actions usually are executed by the execution system, for the most part unconsciously. This is the normal way. However, there can also be unconscious factors influencing this execution in specific, contentual ways. I have found two mechanisms via which this can happen, again priming and psychic resistance.

A famous example of unconscious priming of the execution mode is an experiment by Bargh et al. (1996) revealing the Florida effect: Priming by reading words related to elderliness (e.g., “Florida” and “Bingo”) caused subjects to walk (a little, e.g., speed -11%) slower when they exited the laboratory, compared to subjects who read words that were not related to the elderly (Bargh et al., 1996). In this experiment the priming does not alter the intention – probably there is only the intention to walk to the next station in the course of experiments but no intention to walk slowly or like an elderly person –; the priming alters only the intention’s execution. For many actions the intention is somewhat vague and leaves much leeway to the execution system to fill in the details like speed, trajectory of movements, temporal order of systematically independent steps. In the present example, priming on the stereotype of the elderly may have led to an alteration of the subjects’ psychic atmosphere, which in turn, via a sort of automatic acting out, correspondingly influenced the execution mode.

Another cause of unconscious but characteristic alterations of an action’s execution mode can be inner conflicts. The agent has decided to execute a particular action, however, against (open but sometimes also unconscious) misgivings and personal resistances. This conflict may then alter the execution mode in favor of the personal objections. In particular the resistance may weaken the effort invested in the action, which may be sufficient to make the action inefficient or even ineffective. An example may be that when subjects throw darts at pictures of persons whom they like they achieve a much worse score as when throwing at pictures of neutral persons (Rozin et al., 1986, 705–708; discussed by: Gendler, 2008, 636). Throwing darts at pictures of liked persons is probably seen as a violation of these persons and infidelity with respect to them; imagine what the agent would feel when the respective person entered the room and saw the scene! (Different explanation: Gendler, 2008).

How should unconscious influences on the execution mode be assessed from the practical philosophical standpoints? *C1. Optimality judgment theory of deliberation and intention:* Again the just given explanations are compatible with the optimality belief theory and intentional causalism since these theories require neither that an action be determined in all details by the respective intention – which by the way would be impossible –, nor that the execution mechanism work perfectly. *C2. Agency and intentionality:* In the priming example, the resulting action is clearly covered by the (presumable) intention because this intention leaves sufficient margin for the walking speed: The real speed changes are rather small and can be revealed only by exact measurement and statistics. Hence the subjects walk intentionally. The primed walking speed instead is not intended and, according to the simple view about intentionality (Adams, 1986), is a fortiori not intentional. Analogously, throwing the dart is intentional, hitting the target is intentional, missing it is unintentional since the intention was to hit the target. The resistance, which (let us suppose at least in some cases) is the cause of the misthrow, does not make up an intention, hence cannot render it intentional. Even much more spectacular failures mean that the execution is defective but they do not exclude that the resulting behavior is an action. *C3. Responsibility:* The subjects’ direct responsibility in these examples goes as far as their intentionality goes (R3). So the person who does not hit the picture of the liked person is not directly responsible for this. Is he *indirectly* responsible? Consider this example: A sniper is to eliminate a terrorist with an unexpected shot. The sniper so far has only fired at cardboard targets, but never at living people; he is scrupled and misses his target – contrary to his excellent training results –; the mission fails. In this case the sniper might be indirectly responsible for the failure, namely if he knew about his success-damaging scruples and could have taken alternative measures (e.g., deployment of another sniper, use of target-finding projectiles...). *C4. Freedom:* Freedom of decision is not infringed *per se* by the mechanisms working in these examples, because these mechanisms only operate after the decision has been made. In drastic cases, however, freedom of action could be restricted to the extent that the ability to achieve one’s goals is hampered. Nonetheless, the scruples in the darts example could be an indicator that the decision was not authentic. However, this lack of authenticity would be accidental, because the agent may have consciously reflected his doubts in the decision and nevertheless rationally and authentically decided to throw.

## 9. Unconscious Deliberations and Decisions

The last group of unconsciously influenced actions to be discussed here are unconscious actions based on unconscious decisions – where these unconscious decisions, the preceding deliberations and the resulting intentions are mental states, which are unconscious in the sense of being preconscious (and hence often consciously retrievable) or only reconstructable from known clues (by the subject but sometimes only under professional guidance or only by experts) or accessible by psychoanalytic means. Generally for this group it holds that unconscious deliberation is possible and fairly differentiated, e.g., flexibly adapted to the present situation and includes pointedly and creatively searching for fitting actions. In many respects such completely unconscious deliberations function like conscious ones: possible actions are considered and evaluated according to their consequences and the one with the highest evaluation is executed. But compared to conscious deliberation, unconscious deliberation is primitive. It does not ponder advantages and disadvantages but is simplistically goal-oriented in that finding an effective means may be sufficient for choosing it. So only one positive option and inaction are compared and only one (desired) consequence is taken into account. Moreover, unconsciously deliberated actions are mostly of relatively small size only, partly because only to a limited degree does unconsciousness permit coordination of several small actions. The primitiveness of completely unconscious deliberations is, of course, due to the limited abilities of the unconscious; in particular, due to modularization and the lack of access to the universal workplace, it cannot consider much more alternatives and consequences than just described. In a certain sense, actions caused by completely unconscious deliberations are easier to explain than many previous examples with only some unconscious components, because no contentual influence of unconscious factors on a conscious decision has to be explained. – Apart from these general features, actions based on unconscious decision are a relatively big and heterogeneous group so that several subgroups have to be analyzed separately.

*1. Plain unconsciously deliberated action:* The most basic form of actions based on unconscious decisions are *plain unconsciously deliberated actions*: Elementary deliberations on a sophistication level of Aristotelian practical inferences (“I have aim *e*. My doing *a* is a (sufficient) means to *e*. Therefore, I do *a.*”) are simple enough to be processed unconsciously, however, with the risk of overlooking consciously recognizable problems of the chosen option. There is no dynamic reason for the unconscious processing but only an economic reason, viz. to save the scarce resource of conscious attention. Examples of this kind are: While standing during a conversation the agent supports herself skillfully on the backrest of a chair just standing there; while doing this the agent may be fully concentrated on the conversation. Or a teacher, while being absorbed by his lecture and walking back and forth, may unconsciously open a window. Such actions, while being unconsciously initiated and of modest complexity, probably are not yet automatic routine actions triggered by the perception of a certain stimulus because the situation is too singular for being the trigger of an already learned fitting routine. The advantage of such actions is their efficiency in not using the scarce resources of consciousness; their general disadvantage, however, is the increased risk of overlooking better alternatives and, still more important, significant negative consequences because the action proposal is not exposed to the critical examination of consciousness.

*2. Unconscious decisions with dynamically unconscious motives:* In another subform the unconscious deliberation includes dynamically unconscious motives. The fact that these motives are delicate and, therefore, have been consciously criticized in some way, then suppressed and may again be criticized if getting conscious is the reason why they can express themselves and have successful actional consequences only in unconsciously deliberated actions. Although Freud analyzes many expressions of unconscious influences, in particular Freudian slips and neurotic symptoms, most of them are not really intentional; they do not result from unconscious deliberation and decision but are mainly failures of automatisms due to (sometimes motivated) lack of attention.^9^ However, some of them seem to be intentional actions. A famous example was reported by Freud about himself. When trying to provide a chair for an old person, a young and attractive girl with the same intention arrives at the chair a moment before Freud and takes it. Freud, still having the unaccomplished intention to provide the chair, embraces the girl from behind to take the chair as well, thereby touching the girl’s lap for a moment. He immediately releases the embrace and dissolves the absurd and delicate situation, which nobody seems to have recognized as abnormal. A self-analysis reveals Freud’s unconscious erotic motive to touch the attractive girl at an intimate part of her body (Freud, 1901–1924/1941, 194). This may be analyzed as follows: The overall action, to fetch a chair for the old person, has been consciously deliberated and decided. However, when the events do not occur as planned and a decision how to continue after the rush to the chair has to be taken, Freud unconsciously acknowledges the chance to erotically touch the girl, values it positively and realizes it. Evidence that all this happened unconsciously is that only after the beginning of the erotic action does Freud consciously acknowledge the delicate and prohibited character of his doing and then stops it immediately and consciously.

*3. Sleepwalking:* Typical sleepwalking consists of the sleepwalker getting up from bed, walking through the house open-eyed but with a rigid gaze, e.g., to the toilet, then returning to bed and continuing to sleep. If he is spoken to, he answers briefly but usually incomprehensibly. The sleepwalker is not aware of this and cannot remember this episode after waking. Single photon emission computed tomography of a sleepwalking patient showed deactivation of large areas of frontal and parietal association cortices during the episode, together with activation in the anterior cingulate cortex and thalamus. So the patient was motor-excited, but mentally calm, unconscious (Levy, 2014b, 73–74, referring to Bassetti et al., 2000). In rare cases sleepwalkers are capable of very complex actions – longer car journeys, writing short but mutilated e-mails, even homicides (Levy, 2014b, 71–73; 77). Despite their complexity, the largest parts of these actions are automatic and stimulus-induced. But primitive decisions are made and new action sequences are initiated, as they correspond to the abilities of a mind cut off from further considerations of consequences.

After having analyzed the functioning of several types of unconscious actions based on unconscious decisions let us turn to our practical philosophical questions about them. *C1. Optimality judgment theory of deliberation and intention:* All of these actions conform to the optimality belief theory. The deliberations are unconscious and very simple but still function in the way of searching for advantages and disadvantages of options and aggregating them to optimality judgments – even if only two options with one advantage or disadvantage are considered. And the optimality judgments then function as intentions which cause the corresponding behavior. *C2. Agency:* Consequently, if the unconsciously deliberated optimality judgment causes the corresponding behavior in a non-deviant way then, according to intentional causalism, this behavior is an action in the broad sense and unconsciously intentional. However, it is not an action in the emphatic sense because it is not controlled by the conscious ego and because other values of the subject (than the one strived for via the action’s aim) that could be relevant for the evaluation of the alternatives are not considered in the deliberation (Lumer, 2013). This verdict may seem debatable for *plain* unconsciously deliberated actions because the action mostly corresponds to what the conscious ego could have decided as well. However, this desirable result does not change the underlying structure with its lack of conscious control, which sometimes leads to highly problematic results. Unconscious actions *with dynamically unconscious motives* are still less actions in the emphatic sense since these motives are even rejected by the conscious ego, and the subject would have dismissed the executed option if she had decided consciously. *C3. Responsibility:* Because of their complete unconsciousness, unconsciously deliberated actions fulfill neither the mens rea condition (R3) for responsibility nor that of the soundness of mind (R4). However, plain unconsciously deliberated actions and those with dynamically unconscious motives – unlike sleepwalking – usually take place alongside conscious activities and are monitored to some extent. That is why we often discover that they are taking a negative course and can then consciously interrupt them – as Freud did in the autobiographical example. This leads to a certain indirect responsibility for these actions. *C4. Freedom of decision:* Decisions based on unconscious deliberation are neither autonomous nor authentic nor rational, above all because there is insufficient access to the available information on relevant consequences and to the pertinent evaluation bases, and the ability to integrate greater amounts of information is also lacking.

## 10. Conclusion

Let me recap the results of the preceding discussion.

*C1. Optimality judgment theory of deliberation and intention:* All the considered unconsciously influenced actions are covered by the optimality belief theory; hence this theory has been confirmed.

*C2. Agency:* Furthermore, according to intentional causalism, all the discussed mechanisms represent actions at least in the broad sense. The actions and the goal attainment are intentional, though, in the cases of *deliberation with dynamically unconscious motives* and *entirely unconscious deliberation*, only unconsciously intentional; and with *unconsciously altered execution modes*, the intentionality of the action under certain relevant descriptions may be restricted. Not all of these actions are actions in the emphatic sense (i.e., actions whose deliberation, using the aggregation mode and the critical potential of the conscious ego – and hopefully also respecting standards of rationality –, virtually consider and integrate all of the subject’s values): *Unconsciously deliberated actions* are not actions in the emphatic sense, and particularly so, to an always problematic degree if they are driven by consciously rejected desires; if *conscious decisions* are *influenced by dynamic unconscious motives*, emphatic agency is somewhat curtailed. Intentional causalism as a theory about what actions are has mastered the challenge from unconsciousness well: It captures and classifies differentiatedly all analyzed behaviors which should (not) be classified as actions.

*C3. Responsibility:* We are directly responsible for *consciously deliberated actions* in which the decision is *influenced by unconscious priming or dorsal information*. In the case of *unconsciously altered execution*, direct responsibility extends as far as intentionality, and in most cases quite far. The situation is most complex with *actions based on conscious deliberation influenced by dynamically unconscious motives*; they must be considered in detail in each case: In serious cases (e.g., psychopathologies) the conscious integration of information is so distorted that even soundness of mind, hence responsibility (at the moment) is missing (R4). Apart from that, we are directly responsible for these actions and their consequences as far as the conscious intentionality goes; however, we are not directly responsible for the merely unconsciously intended action characteristics and consequences. Finally, we are never directly responsible for *unconsciously deliberated and decided actions*. In case of lacking direct responsibility there still may be an indirect responsibility for not having taken measures to prevent the loss of direct responsibility; i.e., knowing about all these mechanisms, it could be wise to use stronger measures of self-control or to intervene against excessive *influences of dynamically unconscious motives*; and such measures could also be morally required. We also have some secondary responsibility for actions based on plain unconscious decisions, insofar as we can interrupt them as soon as we realize that they are going in a wrong direction. In such cases some of the analyzed restrictions of direct responsibility would be compensated by an extension of our indirect responsibility.

*C4. Freedom of decision: Influences on conscious deliberations due to unconscious priming* hardly affect freedom of decision (at least in the cases considered here). If the *execution of the action is influenced by unconscious factors*, this does not curtail freedom of decision, because this influence only occurs after deciding, but does in part curtail freedom of action. Rationality may be curtailed when the *conscious decision* is *influenced by unconscious*, but false, too simple *information from the dorsal pathway*. The *influence of dynamically unconscious motives on conscious decisions* more or less severely curtails freedom of decision, because these motives are excluded from being assessed by the ego, so that they cannot possibly be filtered out critically. Finally, *completely unconscious decisions* are generally not free.

Rearranging the just summarized results about responsibility and freedom, one might differentiate three groups of mechanisms:

*A. Unconscious influences on our actions already known before 1980:* 1.2. (This numbering refers to the list at the beginning of section 5). Influence of dynamically unconscious motives on conscious decisions curtails freedom of decision; and these influences can eliminate responsibility entirely or reduce it considerably; but because this mechanism is known and often we can take measures against its influence we can be indirectly responsible for the respective actions. 3.2., 3.3. Our unconscious decisions on the basis of dynamically unconscious motives as well as decisions during sleepwalking are neither free nor are we responsible for them. This group (A) of unconsciously influenced actions contains the severest curtailments of freedom and responsibility; but they are long known, already considered in jurisdiction, and more recent analysis says that targeted influences of dynamically unconscious motives (1.2 and 3.2) are much rarer than Freud assumed.

*B. Unconscious, mostly harmless influences on our actions detected after 1980:* 1.1. Unconscious priming of conscious decisions or 1.3. influences of (wrong) dorsal pathway information on conscious decisions have been recognized only much more recently than mechanisms of group A. Usually they do not curtail responsibility, priming does not curtail rationality and freedom of decision, whereas unconscious influences of dorsal pathway information can restrain rationality and freedom but only slightly. Altogether these influences are harmless. Given the widely expressed worries about curtailments of freedom and responsibility by “the new unconscious,” this result of our discussion is most surprising.

*C. Unconscious, somewhat more critical influences on our actions detected after 1980:* 2.1., 2.2. The execution of actions altered by unconscious priming or psychological resistance leaves the freedom of decision unaffected, but dissolves our direct responsibility for deviations from our intentions. However, even in this case indirect responsibility is possible, if one knows these mechanisms and can work against them. 3.1. Plain unconscious decisions are neither free, nor are we directly responsible for their results; but again we can be indirectly responsible for them. However, and this is reassuring, such plain unconscious decisions usually involve only small actions and they are also somewhat monitored alongside the actions in focus, so that they can be interrupted immediately in critical cases. Group C therefore contains the newly discovered, actually critical unconscious mechanisms. But even they are far less problematic than many voices of public discussion suggest: For they concern only small actions or limit only responsibility, but not freedom of decision; and, with appropriate caution and the use of compensatory techniques, most of the limitation of direct responsibility can be compensated by indirect responsibility.

Altogether then, the more recent (since about 1980) psychological discoveries about unconscious influences on our actions complement our ideas about the functioning of actions. However, for practical philosophical and legal concerns they are much less relevant than originally thought and often stated. They change very little with respect to the question of the agential character of and responsibility for our actions as well as freedom of decision. The most important restrictions in these respects are still those, known for a long time, of somnambulism and dynamically unconscious motives, where the latter, however, have been found to be much less present than Freud thought. This is good news theoretically, because intentional causalism, the standard conception of responsibility and the rationality and the autonomy conception of freedom, i.e., the most important theories in these fields, have thus withstood criticism. And it is also good news practically, because the present practices of attributing responsibility and enhancing freedom of decision do not have to be abandoned and replaced by completely uncertain and worrying alternatives.

## Author Contributions

The author confirms being the sole contributor of this work and has approved it for publication.

## Conflict of Interest Statement

The author declares that the research was conducted in the absence of any commercial or financial relationships that could be construed as a potential conflict of interest.

## Footnotes

1 Freedom of action: Subject *s* is free to do *a*/*s* can do *a*, iff holds: If *s* decides to do *a*, then *s* does *a* (cf. Moore, 1912, ch. 6).

2 Here “ego” does not mean some “homunculus” or a department in the brain, but rather the cohesive mental structure (consisting of unrefused mental events, states, dispositions, and underlying structures) that controls action as an inner subject through the formation of intentions (Lumer, 2014d, section 6).

3 Levy in his outstanding theory (Levy, 2014a,b) also justifies the necessity of consciousness for responsibility with the global workspace. However, he sets another focus, which I would call “autonomy.” I will return to this issue below (in footnotes 5 and 6).

4 Dijksterhuis and co-authors (Dijksterhuis, 2004; Dijksterhuis et al., 2006) have tried to prove the opposite thesis, viz. that, exactly on the contrary, an unconscious integration of the different information leads to better results than the conscious one. However, first, what Dijksterhuis and his co-authors call an “unconcious thought” is a deliberation based on *consciously* received information about the available options and their features; hence, in their experiments only a small part of the deliberation, viz. the integration of that information to a decision, is unconscious. So, in these experiments the importance of consciousness in acquiring that information is not put into question. Second, Dijksterhuis’ experimental situation is designed exactly to queer a good conscious integration of the many pieces of (conscious) information: Subjects are bombarded with a huge mass of data in a very short time, e.g. 48 features in random order for four seconds each. During the following three minutes, dedicated to conscious reflection, it is impossible to recall all these features and then to consciously count advantages and disadvantages of the options or to compare all options with respect to the single dimensions in descending order of importance (cf. Payne et al., 1993). With a well ordered written list of the features and sufficient time, conscious decision would obviously be much more precise than the unconscious deliberation. Third, in most of the examples the advantages and disadvantages had only to be counted but not weighted. Since unconscious quantitative impression formation on consecutively experienced items works quite well, the examined way of (additive) information integration was quite favorable for unconscious processing (and much less for conscious processing of mostly no longer present information). This will probably be quite different with decisions in which the various features have diverse importance for the subject and the subject has time to systematize the obtained information. – Further critique: Levy, 2008; Levy, 2014b, 58–62.

5 Reflections of this kind play the leading role in Levy’s theory and are further developed by him (Levy, 2014a,b). However, Levy does not regard this kind of integration of all important concerns of the subject into the decision to act only as a condition of *autonomy* and freedom of decision, but already as a condition for *responsibility*.

6 Michael Moore also sees consciousness of the decision and of the relevant consequences as necessary conditions for responsibility, because in the case of unconsciousness the unity of consciousness is not given and there is no access to relevant desires and beliefs (Moore, 1980, 1620–1621; Moore, 1984, 339–342; Moore, 1993/2010, 258). This is correct, but remains somewhat dark in detail. – Levy, instead, in his important study justifies the need of consciousness for responsibility, as it is done here, with the need for access to the global workspace. But for him this access is necessary in order for the action to be connected to the person, to be consistent and coordinated with other attitudes; the attitudes behind the action belong to the true self only if they are part of a coherent perspective from which the options can be evaluated (Levy, 2014a; Levy, 2014b, 87–108). However, this justification sets the conditions for responsibility too high; they are conditions for an *autonomous* and *authentic* decision, but not just for responsibility. The conditions for freedom of decision are stronger than those for retrospective responsibility. The latter are part of the system of social control of action and are mainly to guarantee the subject’s effective and efficient control of her action (Lumer, 2012, 708–713); however, this controllability is not only given with a rather ideal freedom of decision. Accordingly, also a judge will not be impressed by the inner “strife” of a person as the background of some deed, e.g., between the life of a family father and that of an artist – I am not talking about clinical cases of schizophrenia – when it comes to punishing a serious crime.

7 For example: “At the time of the committing of the act, the party accused was laboring under such a defect of reason, from disease of the mind, as not to know the nature and quality of the act he was doing; or if he did know it, that he did not know he was doing what was wrong” (M’Naghten rule). “Who, when committing the act, is unable to recognize the wrong of the act or to act according to this insight because of a pathological mental disorder, because of a profound disturbance of consciousness or because of imbecility or a severe other mental anomaly, acts without guilt” (German penal code: Strafgesetzbuch §20).

8 Bargh and his co-authors explain this result by an unconscious activation of an achievement goal, which then again unconsciously led to a better performance [“auto-motive hypothesis” (Bargh et al., 2001, 1014–1016; 1024; see also: Chartrand and Bargh, 1996, 465)]. I find this little plausible. First, achievement *goals* are rather specific; but when the subjects are working on the first task they do not know anything about the second task and about what higher achievement in it could consist in. Therefore, the achievement priming may have activated an achievement *desire* but not yet an achievement *goal*. Second, high achievement in the second task requires unwaveringly following a systematic strategy. This probably is not possible without consciously following this strategy, which in turn presupposes some conscious decision to apply this strategy.

9 Analyses of action slips as failures of automatisms: Reason and Mycielska (1982).
